# Chocolate-based Ink Three-dimensional Printing (Ci3DP)

**DOI:** 10.1038/s41598-019-50583-5

**Published:** 2019-10-02

**Authors:** Rahul Karyappa, Michinao Hashimoto

**Affiliations:** 1 Digital Manufacturing and Design (DManD) Centre, Singapore University of Technology and Design, 8 Somapah Road, Singapore, 487372 Singapore; 2 Pillar of Engineering Product Development, Singapore University of Technology and Design, 8 Somapah Road, Singapore, 487372 Singapore

**Keywords:** Colloids, Rheology

## Abstract

Recent advances in three-dimensional (3D) printing technology has enabled to shape food in unique and complex 3D shapes. To showcase the capability of 3D food printing, chocolates have been commonly used as printing inks, and 3D printing based on hot-melt extrusion have been demonstrated to model 3D chocolate products. Although hot-melt extrusion of chocolates is simple, the printing requires precise control over the operating temperature in a narrow range. In this work, for the first time, we directly printed chocolate-based inks in its liquid phase using direct ink writing (DIW) 3D printer to model complex 3D shapes without temperature control. We termed this method as chocolate-based ink 3D printing (Ci3DP). The printing inks were prepared by mixing readily available chocolate syrup and paste with cocoa powders at 5 to 25 w/w% to achieve desired rheological properties. High concentrations of cocoa powders in the chocolate-based inks exhibited shear-thinning properties with viscosities ranging from 10^2^ to 10^4^ Pa.s; the inks also possessed finite yield stresses at rest. Rheology of the inks was analyzed by quantifying the degree of shear-thinning by fitting the experimental data of shear stress as a function of shear rate to Herschel-Bulkley model. We demonstrated fabrication of 3D models consisting of chocolate syrups and pastes mixed with the concentration of cocoa powders at 10 to 25 w/w%. The same method was extended to fabricate chocolate-based models consisting of multiple type of chocolate-based inks (*e*.*g*. semi-solid enclosure and liquid filling). The simplicity and flexibility of Ci3DP offer great potentials in fabricating complex chocolate-based products without temperature control.

## Introduction

This paper describes an approach to perform 3D printing of chocolate-based materials in its liquid phase at room temperature, termed chocolate-based ink 3D printing (Ci3DP). We printed the chocolate-based inks in the form of chocolate syrup and chocolate paste mixed with varying concentrations of cocoa powders. The rheology-modified chocolate-based inks exhibited viscous and Herschel-Bulkley fluid properties, and they were printable using a DIW 3D printer without temperature control. Ci3DP is complementary to currently available methods of 3D printing of chocolates based on hot-melt extrusion where the printing ink is heated and molten before patterning. Wide selection of chocolate-based materials, and ability to tailor their properties, distinguish our current approach as a unique method to 3D print chocolate-based products at room temperature.

Recent advance in digital fabrication, in particular 3D printing, has enabled free-form fabrication of 3D structures and devices from plastics, metals, and other raw materials. To date, 3D printed structures have demonstrated a wide range of applications including sensing^1,2^, tissue engineering^3,4^ and physiological vascular modelling^5,6^. Recent frontier of 3D printing has focused direct printing of edible materials^7–9^. 3D food printing allows printing of foods with customized contents of nutrients, where the nutrients can be, in principle, optimized based on biometric and genomic information^10^. Similarly, to other 3D printing techniques, 3D food printing relies on layer-by-layer fabrication based on the CAD data and sequential depositions of 2D layers. The food inks can be customized by concerted combination of the essential ingredients of food such as carbohydrates, proteins and fat. Different 3D printing techniques, selective laser sintering (SLS), ink-jet printing, hot-melt extrusion and binder jetting, have been demonstrated for 3D food printing^11^. Amongst all, extrusion is the most popular method in 3D food printing. During extrusion, materials (which are liquid, semi-solid or solid) are forced through a nozzle to fabricate 3D objects. There are three types of extrusion techniques: cold, hot-melt and gel-forming extrusion^12^. Cold extrusion is performed at room temperature without phase change. Hot-melt extrusion is primarily based on the mechanism similar to fused deposition modeling (FDM)^13^: (1) heating to flow the ink out of the nozzle and (2) cooling to solidify molten chocolates in the bed. Gel-formation extrusion involves phase change after extrusion of liquid materials. Techniques based on extrusion have limitations with respect to the stability and self-supporting properties of the printed objects^11^. Nevertheless, owing to their simplicity, extrusion has been used to print different types of foods^14^.

Chocolate, a complex emulsion of fine solid particles of sugar and cocoa in a continuous fat phase^15^, is a universally loved food. As a material, chocolates are solid at room temperature (20 to 25 °C), and melt in human mouth and saliva (37 °C)^16^. The melting characteristics and the texture of chocolates depends on the types and proportions of cocoa and other ingredients, and the methods to blend and process them^17^. The components are mixed, refined and conched during processing so that a suitable viscosity of chocolate is obtained prior to tempering^17^. Chocolates are commonly used for the demonstration of 3D food printing because it can be patterned by hot-melt extrusion and it becomes solid at room temperature. Several studies related to hot-melt extrusion of chocolate are available in the literature^13,18–21^. In the reported experiments, the chocolate was melted and dispensed at the temperature of 31 to 36 °C; the attention to temperature was required because the rheological characteristics of the molten chocolate was sensitive to temperature. Hot-melt extrusion of chocolate offers advantages in simplicity and accessibility, while there is a drawback inherent to its printing mechanism; the control over the narrow range of operating temperature is required. Cold extrusion depends solely on the rheology of printing ink at the operating temperature^11^. The manipulation of temperature is not required, and the entire printing can be performed at room temperature. 3D modelling of chocolate-based materials by cold extrusion has not been well-studied to date, however, due to the lack of inks possessing suitable rheological properties.

To bridge this gap, we developed a method to print chocolate-based inks at room temperature by cold extrusion. In this study, ‘chocolate-based inks’ are defined as liquid chocolate products mixed with cocoa powders; readily available chocolate products (*i*.*e*. syrups and pastes) were mixed with cocoa powder to alter the rheology of the ink. The rheological properties of the chocolate-based inks were studied to identify the properties suitable for DIW at the room temperature. To obtain good print fidelity, the effects of the three parameters of the printing—applied pressure (*P*), deposited mass of the ink per unit length (*m*) and the distance between two layers (*Δz*)—were studied. Under the conditions optimized for printing, we demonstrated to create 3D models using a single and multiple chocolate-based inks. Ci3DP is capable of fabricating 3D structures at room temperature, and the principles and methods presented here should expand the toolkit of 3D food printing and should be applicable to 3D print other edible inks by a DIW printer.

## Results

### Overview of the study

**Figure 1:**
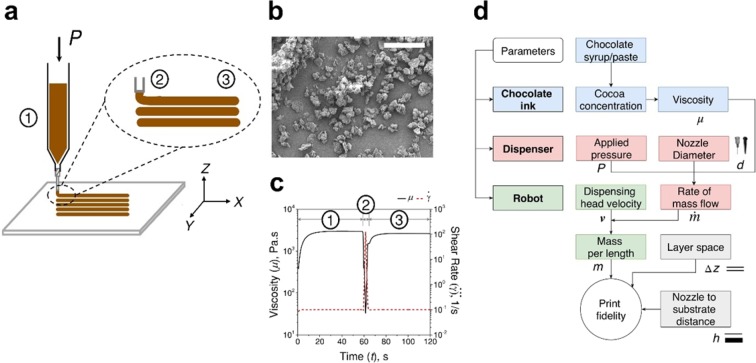
Concepts of chocolate-based ink 3D printing (Ci3DP). (**a**) Schematic illustration of DIW of chocolate-based inks at room temperature. Shear-thinning flow of the ink facilitated easy extrusion through the syringe (Region 1) and the nozzle (Region 2). The ink exhibited fluid with yield stress behavior to form self-supporting layers after extrusion (Region 3). (**b**) SEM micrograph of cocoa powder used to prepare chocolate-based inks in Ci3DP. (**c**) Representative flow of chocolate-based ink during extrusion through syringe (Region 1), during extrusion through nozzle (Region 2), and after extrusion through nozzle (Region 3) by measuring *μ* with the change in $\dot{\gamma }$ by rheometry. (**d**) Parameters in Ci3DP to be considered for printing. Scale bar = 50 μm.

The general scheme of Ci3DP is shown in Fig. 1(a). Chocolate-based inks were formulated by mixing syrup and paste with cocoa powder (Fig. 1(b)). We evaluated the printability of the chocolate-based inks in two terms: (1) rheological properties of the chocolate-based inks and (2) parameters of DIW 3D printing. The rheological properties of the formulated inks determined the suitability of the inks to perform cold extrusion. The formulated inks must exhibit shear-thinning behavior for easy extrusion through the syringes and nozzles, and form self-supporting layers after extrusion to maintain the printed structures (Fig. 1(c) and Supplementary Information). We confirmed that the dimension of the printed inks were maintained without spreading when the ink contained high concentration of cocoa powders. Commercially available cake icing was used without modification as a reference of a printable material with suitable rheological properties. Thereafter, the parameters of 3D printing were identified (Fig. 1(d)). A DIW 3D printer consisted of a dispenser and a motion control robot (Fig. S1). We identified the operating parameters for the successful 3D modelling of chocolate-based inks to ensure proper attachment between printed layers, and demonstrated fabrication of 3D models consisting of chocolate-based inks without temperature control.

### Parameters in Ci3DP

Successful Ci3DP required the chocolate-based ink with adequate rheological properties, and the use of the right setting of the instrument. In brief, DIW 3D printing have three different elements requiring optimization: ink, dispenser, and robot (Supplementary Fig. S1). Firstly, the printing ink must exhibit suitable rheological characteristics. We discuss in the next section that yield stress (*σ*_*y*_) and the storage modulus (*G*′) of the ink were the most crucial parameters that governed the outcomes of printing. Secondly, the dispenser (consisting of the pressure source, syringe and nozzle) determined the rate of mass flow dispensed from the nozzle. The nozzle attached to the syringe provided high fluidic resistance to the viscous fluid flowing through it. The applied pressure drop (*ΔP*) and the diameter of the nozzle (*d*) governed the rate of mass flow through the given nozzle ($\dot{m}$). Finally, the motion-control robot (attached to the syringe and the nozzle) controlled the movement of the syringe during the deposition of the ink. The velocity of the dispensing head (*i.e.* syringe and nozzle) (*v*) determined the mass of the ink dispensed per unit length (*m* = $\dot{m}/v$). The robot also offered the control over the motion in the vertical direction (*i.e.* perpendicular to the layer of printing). When the 3D model is created layer by layer, the distance between adjacent layers (*Δz*) and the nozzle-to-substrate distance (*h*) were considered to achieve good print fidelity (Supplementary information). We summarized the relationship between the investigated parameters (Fig. 1(d)).

### Rheological characterization of chocolate-based inks

Initially, we characterized the chocolate-based inks for viscosity (*μ*), yield stress (*σ*_*y*_) and recovery behavior. The inks were prepared by adding cocoa powders to chocolate syrup (10 to 25 w/w%; 10 w/w % is denoted as S10 etc) and to chocolate paste (5 to 12 w/w%, 5 w/w% is denoted as P5 etc). The measurements of *μ* as a function of shear rate ($\dot{\gamma }$) are presented (Supplementary Fig. S2). For the range of the concentrations we explored, the addition of cocoa powder increased *μ* by 10^4^ for chocolate syrups (10^1^ Pa.s for S0 and 10^4^ Pa.s for S25) and by 10^2^ for chocolate pastes (~800 Pa.s for P0 and 10^4^ Pa.s for P12). Previous studies have shown that the inks with *μ* > 100 Pa.s were printable by a DIW 3D printer^22,23^. The viscosity of the inks decreased as $\dot{\gamma }$ increased, suggesting the shear-thinning behavior. This property was desirable for DIW because the ink needs to be dispensed through narrow nozzles at the pressure provided by the dispenser.

**Figure 2:**
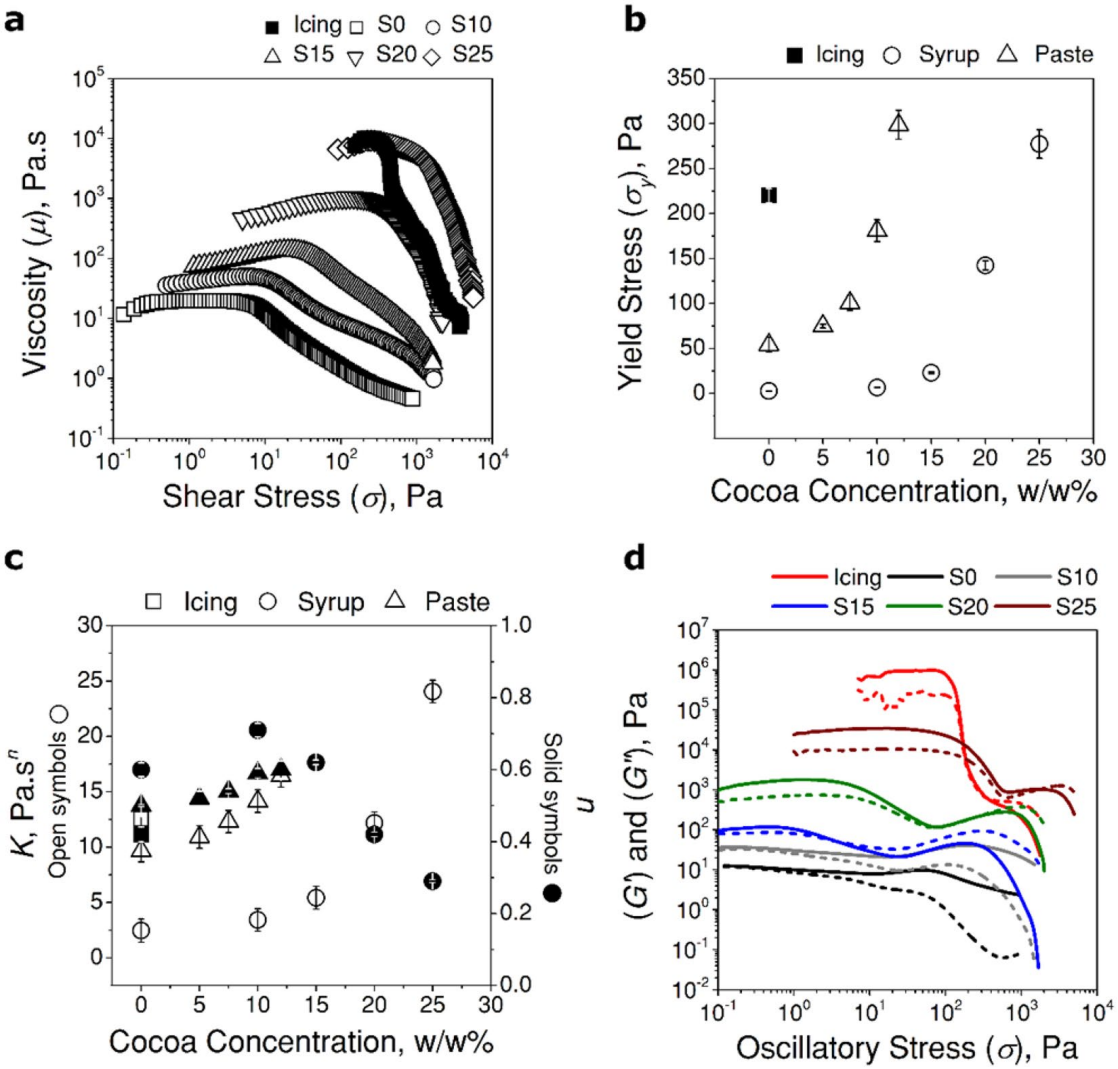
Rheological characterization of syrup (S0), syrup with cocoa powder (S10 to S25), paste (P0), paste with cocoa powder (P5 to P12) and cake icing. (**a**) Viscosity (*μ*) as a function of applied shear stress (*σ*). (**b**) Yield stress (*σ*_*y*_) measured from (**a**) as a function of concentration of cocoa in the inks. (**c**) Parameters of Herschel-Bulkley model (*K* – open symbols and *n* – solid symbols) as a function of concentration of cocoa in the inks. (**d**) Storage (*G*′, solid lines) and loss moduli (*G*′′, dashed lines) as a function of applied oscillatory shear stress (*σ*) for chocolate syrups (S0 to S25) and cake icing.

An important insight into printability is provided by *σ*_*y*_ of the inks. To determine *σ*_*y*_ of the inks, a shear stress ramp was performed; *σ*_*y*_ was analyzed by observing *μ* at which the ink started flowing. As shear stress (*σ*) was gradually increased, sudden decrease in *μ* suggested yielding of the fluid (Fig. 2(a) for chocolate syrup and Supplementary Fig. S3(a) for chocolate paste). *σ*_*y*_ was determined using the intersection point of two tangents—one in the plateau-region of *μ* where the ink deformed elastically and one in the region where *μ* dropped and the ink started to flow (Supplementary Fig. S3(b)). The van der Waals interactions between the colloidal particles were broken once *σ* exceeded *σ*_*y*_. The values of *σ*_*y*_ was measured as a function of the concentration of cocoa (Fig. 2(b)). The measurement suggested that the addition of the cocoa powder increased *σ*_*y*_ from 0 (S0) to 277 ± 22 Pa (S25) for the chocolate syrup, and from 54 ± 8 (P0) to 298 ± 16 Pa (P12) for the chocolate paste. The increase in *σ*_*y*_ suggested that the colloidal network within the ink was reinforced by high cocoa concentration. As a reference material, *σ*_*y*_ for unmodified cake icing was measured to be 220 ± 8 Pa. The cake icing forms self-supporting layers (as they are used to create 3D decorations on cakes), and our measurement verified that formulated chocolate-based inks possessed the same order of *σ*_*y*_ as cake icing. The high values of *σ*_*y*_ guaranteed the inks to maintain the printed structures once printed.

### Shear-thinning behaviors of the ink

It is desirable that the printing ink possess shear-thinning properties to dispense the inks at low pressure in DIW 3D printing. We confirmed that the formulated chocolate-based inks exhibited shear-thinning flow behaviors. The behaviors of the ink was approximated by the Herschel-Bulkley (HB) model^24^:

1 $$\sigma ={\sigma }_{y}+K{\dot{\gamma }}^{n}$$

2 $${\log }_{10}(\sigma -{\sigma }_{y})={\log }_{10}K+n\,{\log }_{10}\dot{\gamma }$$

where *K* is the flow consistency index (a measure of the viscosity of the fluid in Pa.s^*n*^), and *n* is the shear-thinning index (*n* = 1 for Newtonian fluids, *n* < 1 for shear-thinning fluids and *n* > 1 for shear-thickening fluids). The values of *K* and *n* were determined by plotting (*σ* − *σ*_*y*_) with respect to $\dot{\gamma }$ on the logarithmic axes and fitting a straight line in the form of Eq. (2) (Supplementary Fig. S4)^25^. The HB model provided a fit with *R*^2^ > 0.95 for all inks we tested in the experiment. The calculated values of *K* and *n* are shown (Fig. 2(c)). For the cake icing, the value of *n* was 0.4, confirming the shear-thinning property. With addition of cocoa powder in syrup, *n* decreased from 0.7 (S15) to 0.3 (S25) as the cocoa concentration was increased (Fig. 2(c), solid circles). For the inks based on the chocolate paste, slight increase in *n* from 0.5 (P0) to 0.6 (P12) was observed as the cocoa concentration was increased. The lower *n* suggested the higher degree of shear-thinning of the ink. The value of *K* increased as the cocoa concentration increased for both syrup and paste. Overall, the high values of *K* for the chocolate-based inks suggested improved mechanical strength to retain shapes after being dispensed from the nozzle^26^.

The oscillatory amplitude sweep tests were performed to study the viscoelastic properties of the printing inks (Fig. 2(d) and Supplementary Fig. S5(a)). Oscillatory amplitude sweep tests provided details of the microstructure of the inks. The response of storage modulus (*G*′, a measure of elastic response of the ink) and loss modulus (*G*′′, a measure of viscous response of the ink) with applied sinusoidal oscillatory shear was observed. Further, oscillatory stress sweeps were used to analyze the yield behavior of the inks. At low shear stress, *G*′ for all the inks remained constant, and a linear viscoelastic region (LVR) was observed (except for S0 and S10 which showed liquid-like behaviors, which were not suitable to fabricate 3D models). In this region, the internal structure of the inks remained unchanged or undamaged due to their elastic behavior. The solid-like behavior of the inks in LVR was confirmed for the condition of *G*′ > *G*′′. *G*′ in LVR gradually increased with increasing concentration of cocoa powder in the inks, suggesting the presence of strong interactions between the colloidal particles (Supplementary Fig. S5(b)). For the inks formulated with the syrup, *G*′ increased by five orders from S0 to S25. For the inks formulated with paste, the general behaviors were the same as those with the syrup; *G*′ ranged from 10^4^ Pa (P0) to 10^6^ Pa (P12). The broad LVR of the inks with high concentrations of cocoa powders (S20, S25, P10 and P12) indicated high resistance to the applied stresses to maintain the structures (Supplementary Fig. S5(b)). These inks withstood applied oscillatory stress of 10^1^ to 10^2^ Pa before the structure breakdown occurred.

The gradual decrease in *G*′ indicated collapse of the ink structure and subsequent breakdown resulting in liquid-like behaviors. To gain insights into the liquid-like and solid-like behaviors of the inks, we estimated yield stress by two ways: (1) deviation of *G*′ from LVR, measuring the yield point (*σ*_1_) that indicated the onset of fall in the value of *G*′, and (2) crossover of *G*′ and *G*′′, measuring the yield point (*σ*_2_) that indicated the transition from solid-like to liquid-like behaviors^27^ (Supplementary Fig. S5(c)). *σ*_1_ was determined from the intersection of two lines: one fitted to the values in the LVR and another fitted to *G*′ beyond the LVR^28^. As expected, cake icing exhibited large values of *σ*_1_ = 132 ± 10 Pa and *σ*_2_ = 353 ± 25 Pa. Based on the measured values of *σ*_*1*_ and *σ*_2_, the unmodified syrup (S0) was more liquid-like than the unmodified paste (P0) (Supplementary Fig. S5(c)). The values of *σ*_1_ and *σ*_2_ increased with increasing concentration of cocoa (Supplementary Fig. S5(c)). The difference in the values of *σ*_1_ and *σ*_2_ was less for the inks based on the paste than those based on the syrup, suggesting fast solid-liquid transition. Both *G*′ and *G*′′ of the rheology-modified syrups and pastes exhibited overshoots and following decreases at intermediate strain amplitude (Supplementary Fig. S5(d)). This observation suggested Type IV (strong strain overshoot) large amplitude oscillatory shear (LAOS) behavior, a characteristic of strong intermolecular interactions of the network microstructure^29^. For cake icing, only *G*′′ showed an overshoot at intermediate strain amplitude followed by decreasing values suggesting Type III (weak strain overshoot) LAOS behavior^29^ (Supplementary Fig. S5(d)). Such behaviors may be attributed to a transition from an ordered structure to a disordered structure (*i*.*e*. breakdown of the microstructures) that occurs during shearing of the suspensions^29–31^. Further investigation will be needed to understand the physical mechanism of the complex behavior of strain overshoot observed for the studied inks.

Finally, we performed thixotropic loop tests to study the time-dependent breakdown and recovery of the microstructure of the printing inks. The results of the experiments are summarized (Supplementary Fig. S6 and Supplementary Information). We confirmed that degree of thixotropy increased by the addition of the cocoa powder. The thixotropic loop area increased from S0 (19 ± 3 Pa/s) to S25 (5186 ± 462 Pa/s) for the syrup, and P0 (435 ± 47 Pa/s) to P25 (5531 ± 562 Pa/s) for the paste, respectively (Supplementary Fig. S6(b)). Similarly, thixotropic loop area of cake icing was measured to be 889 ± 65 Pa/s. These measurements suggested that the syrup and the paste acquired characteristics of a thixotropic fluid by the addition of cocoa powders.

Overall, we studied the rheological properties of the chocolate-based inks by the addition of the cocoa powders. The ideal ink for DIW 3D printing should be shear-thinning for the ease of dispensing, and should possess high *σ*_*y*_ and *G*′ to retain the resolution of the printed structures without spreading. Overall, previous studies suggested the values of *σ*_*y*_ and *G*′ for successful DIW 3D printing; inks with *σ*_*y*_ ~ 10^2^ to 10^3^ Pa and *G*′ ~ 10^3^ to 10^4^ Pa resulted in good fidelity of printing^32–34^. The fidelity of printing was not confirmed by previously reported rheological characterization of the printing inks. For example, in the study by Lille^34^, it was reported that the ink with *σ*_*y*_ = 60 Pa and *G*′ = 500 Pa was able to retain the printed shape after extrusion. Two cases were reported for the failure of printing; the ink with *σ*_*y*_ = 100 Pa and *G*′ = 36000 Pa experienced the difficulty of extrusion though the nozzle, and the ink with *σ*_*y*_ = 8 Pa and *G*′ of 300 Pa could not retain the printed shape^34^. By comparing the values of *σ*_*y*_ and *G*′ of the chocolate-based inks to that of reported values, S20, S25, P10 and P12 appeared to be suitable inks for Ci3DP. We also note that the formulated chocolate-based ink possessed similar rheological properties to cake icing, a material known to form self-supporting 3D structures.

### Flow of an ink through a nozzle

**Figure 3:**
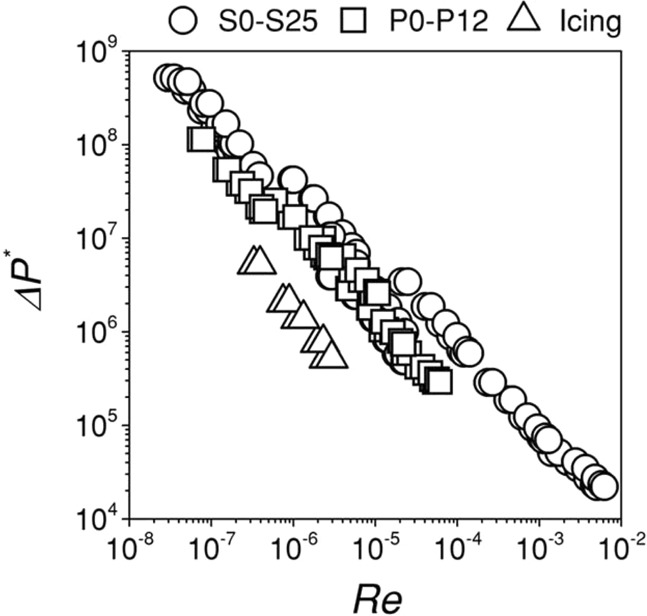
A plot showing the dimensionless pressure drop (ΔP*) as a function of Reynolds number (*Re*) for inks based on chocolate syrups (S0 to S25), inks based on chocolate pastes (P0 to P12) and cake icing. General-purpose nozzles of sizes *d* = 410, 600 and 810 μm was used.

The chocolate-based ink was extruded through the nozzle under an applied pressure (*ΔP* = *P* − *P*_*atm*_) in Ci3DP. The extrusion of the inks through the nozzles influence the process-related rheological properties; the process-related *μ* is lower than *μ* measured by rheometry^35^. It was therefore important to estimate the ink properties from the measurements relevant to the actual process. First, we quantified the relationship between the rate of volumetric flow of the inks (*Q*) and *ΔP* for S0 to S25, P0 to P12 and cake icing with the nozzles of *d* = 410 to 840 *μ*m (22 to 18 Gauges) for *ΔP* = 100 to 550 kPa. The measurements confirmed that, for the given ink, *Q* increased nonlinearly with increasing *ΔP* and with increasing *d* (Supplementary Fig. S7). The high concentration of the cocoa powder resulted in high *μ* and hence low *Q*. The trends observed in the plots of *Q* as a function of *ΔP* allowed to estimate the required pressure to achieve adequate flow rate to model 3D structures by Ci3DP. We used two dimensionless groups to characterize the flow of the inks through the nozzle: the Reynolds number (*Re*),

3 $$Re=\frac{\rho {v}_{i}d}{\mu }$$

and the dimensionless pressure drop (ΔP*),

4 $$\Delta {P}^{\ast }=\frac{\Delta P}{\rho {v}_{i}^{2}/2}$$

where *d*, *ρ* and *v*_*i*_ are the diameter of the nozzle, the density of the ink and the average velocity of the ink through the nozzle, respectively. The plot of ΔP* against *Re* for all the inks exhibited similar linear trends with some scatter (Fig. 3). ΔP* decreased inversely with *Re*; this observation suggested the characteristic viscous-dominated flow^36^, where viscous effects dominate over inertial effects of chocolate-based inks at small *Re* (*Re* < 1).

### Influence of types of nozzles on *Q* of inks

We used two types of nozzles in Ci3DP, a general-purpose nozzle and a tapered nozzle (Supplementary Fig. S8(a)). The resistance of a tapered nozzle was lower than that of a general-purpose nozzle with the same diameter of the outlet; a general-purpose nozzle has a choke point that would prevent or lower the flow of viscous inks. We compared the nozzles with *d* = 600 *μ*m (at the exit area); we dispensed P10 for *ΔP* = 100 to 550 kPa and measured *Q* (Supplementary Fig. S8(b)). For a given *ΔP*, *Q* of the ink in a tapered nozzle was larger by 2 to 3 times that the values of *Q* in a general-purpose nozzle with the same inner diameter of the outlet. For a general-purpose nozzle, more than 90% of the total pressure drop occurs in the last 40% of the length of the nozzle^36^. In contrast, for a tapered nozzle, more than 90% of the total pressure drop occurs in less than the last 20% of the length of the nozzle^36^. The use of tapered nozzle ensured a smooth flow of the ink, and lowered the required pressure applied by the dispenser for the high viscous inks such as S25 and P12. While *ΔP* is the printing parameter most convenient to vary to adjust *Q*, the change in the nozzle type (*i*.*e*. a general-purpose nozzle or a tapered nozzle) also allowed to vary *Q* for the fixed value of *ΔP*. In practice, the use of tapered nozzles was preferred because the printing was performed at low pressure.

### Spreading of printed inks

**Figure 4:**
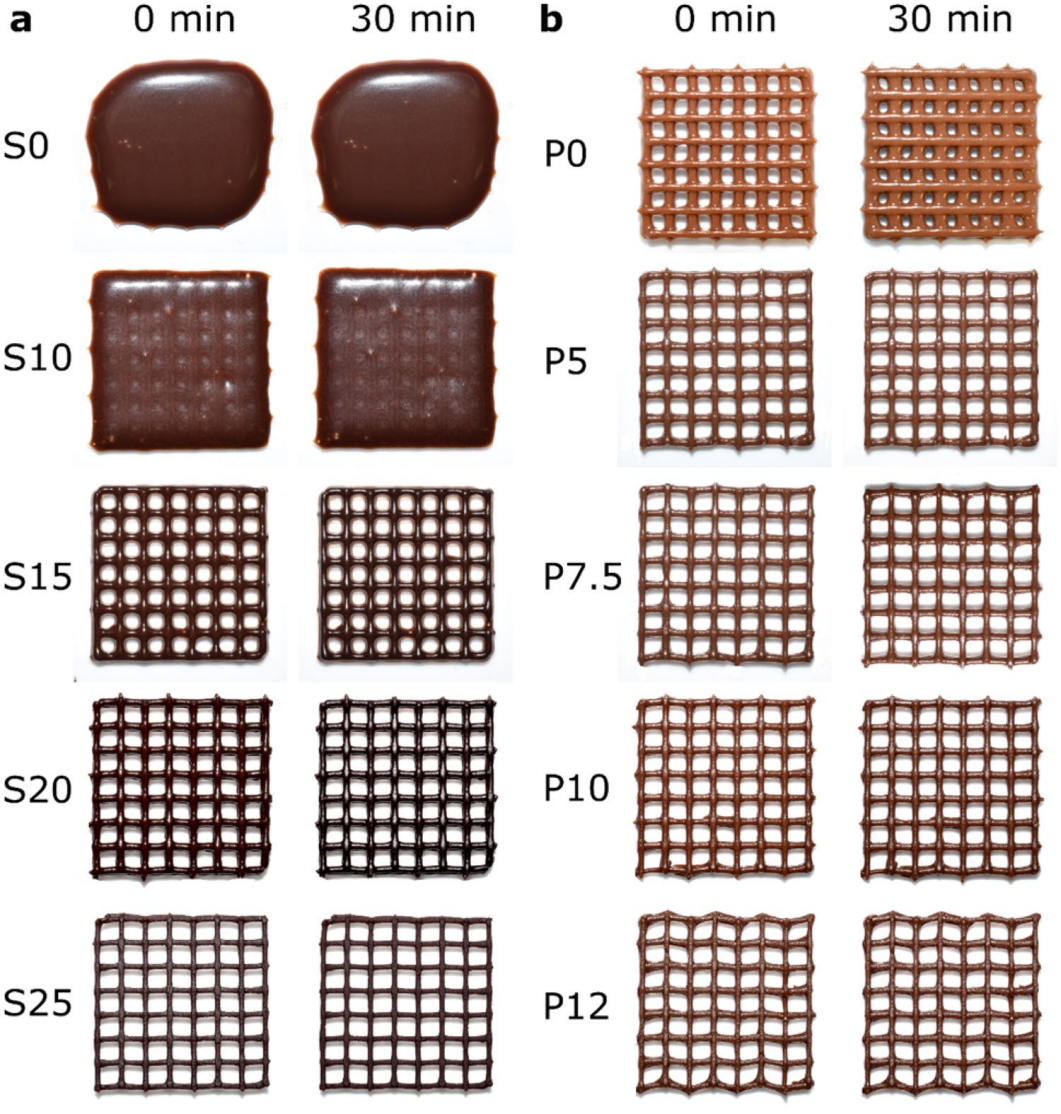
Effect of concentration of cocoa in the chocolate-based inks on the fidelity of printing, highlight the spreading of the inks. The 3D meshes (4 cm × 4 cm, 4 layers) consisting of chocolate-based materials were printed. 3D printed meshes of (**a**) syrup-based inks (S0 to S25), and (**b**) paste-based inks (P0 to P12) are shown at two timepoints: 0 min (immediately after printing) and 30 min after printing.

The printed structure may be affected due to spreading of the inks at time *t*_*p*_, where *t*_*p*_ is the time after printing. Spreading of the ink is pronounced for the inks with low yield stress (*σ*_*y*_). We highlighted this problem by printing a 3D mesh (4 cm × 4 cm) with four layers of chocolate-based inks based on the syrups (S0 to S25) (Fig. 4(a)) and those based on the pastes (P0 to P12) (Fig. 4(b)). We observed that the printed S0, S10 and S15 started spreading immediately while printing (Supplementary Movie S1). *σ*_*y*_ (2 to 23 Pa for S0 to S15) and *G*′ (113 ± 7 Pa for S15) of the ink were not sufficiently high, and the printed structure yielded to the gravity (the magnified images in Supplementary Fig. S9). In contrast, S20 (*σ*_*y*_ = 142 ± 5 Pa, and *G*′ = 1670 ± 115 Pa) and S25 (*σ*_*y*_ = 277 ± 22 Pa, and *G*′ = 35000 ± 3000 Pa) retained the shape of the printed inks for 30 min (Supplementary Movie S2). The large values of *σ*_*y*_ and *G*′ for S20 and S25 allowed the inks to form self-supporting layers. Similarly, we fabricated 3D meshes consisting of four layers of the inks formulated with the chocolate pastes (P0 to P12) (Fig. 4(b)). The values of *σ*_*y*_ were slightly higher than those for the inks formulated with syrups. The spreading of the printed inks was observed for P0; the width of the printed ink increased over 30 min. The width of the filament remained nearly constant for P5, P7.5, P10 and P12. Interestingly, the adjacent layers vertically fused at the locations in contact for the models fabricated in P5 and P7.5, while the different layers of the models consisting of P10 and P12 remained disjoint for 30 min after printing (Supplementary Fig. S9). These experiments highlighted the importance of non-Newtonian rheology of the printing inks on the fidelity of printing. Spreading of the printed structures were avoided by adding large amount of the cocoa powders to the syrup (20 to 25 w/w%) and to the paste (10 to 12 w/w%). The rheology of printing also affected the vertical attachment of different layers, which needs to be ensured to have 3D models.

### Shear-thinning inks and print fidelity

**Figure 5:**
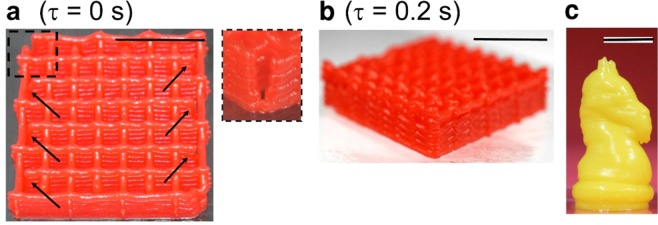
Formation of gaps by printing shear-thinning inks. (**a**) Formation of gaps due to time lag associated with the dispensing of highly viscous, shear-thinning cake icing. The arrows indicate the locations of the gaps, corresponding to the points where the dispensing of the ink started. (**b**) Sealing of gaps. The nozzle was stopped (while *P* was maintained) for 0.2 s at every point to start dispensing the ink. (**c**) A 3D printed knight of cake icing. Scale bars = 1 cm.

The attainable 3D models also depended on shear-thinning property of the inks. We discussed earlier that the chocolate-based inks and cake icing were yield stress and shear-thinning fluids. For such fluids, there are some time lags before the terminal volumetric flow rate (*Q*) was achieved after a given pressure was applied. These time lags need to be compensated to attain proper 3D models. In this section, we used cake icing as a model ink to understand the behavior of the printed inks; the same observations should be applicable for other types of chocolate-based inks. The paths to dispense two layers of the mesh are shown (Supplementary Fig. S10). We observed the gaps in the printed models at the points where dispensing was started (*i.e.* the top left corner of the mesh) (Fig. 5(a)). The attachment of filaments at the corner was compromised and the gaps were formed (Fig. 5(a), inset). These gaps formed due to the time lag associated with the dispensing of viscous shear-thinning solution (*μ* = 2 × 10^4^ Pa.s for cake icing). The ink did not attain the terminal volumetric flow rate (*Q*) immediately after applying the constant pressure for dispensing, resulting in incomplete sealing at the corners of the model. To compensate for this time lag, we stopped the motion of the nozzle for some time (*τ*) at every point where the dispensing started (*e*.*g*. *τ* = 0.2 s used for cake icing). Intended 3D models were obtained with the compensation of time (Fig. 5(b)). Generally, inks with large values of *μ* required to account for the time lag; S20, S25, P10 and P12 (*μ* ~ 10^3^ to 10^4^ Pa.s) required *τ* = 0.2 to 0.5 s to achieve the best print fidelity. In contrast, S0, S10 and S15 did not require such compensation, and *τ* = 0 was used for printing (although the printed models failed due to spreading of the inks). This consideration allowed fabricating relatively complex structures of cake icing at room temperature by DIW; a 3D object representing a knight was fabricated using cake icing with proper attachment of the printed inks (Fig. 5(c)).

### 3D structures by Ci3DP

**Figure 6:**
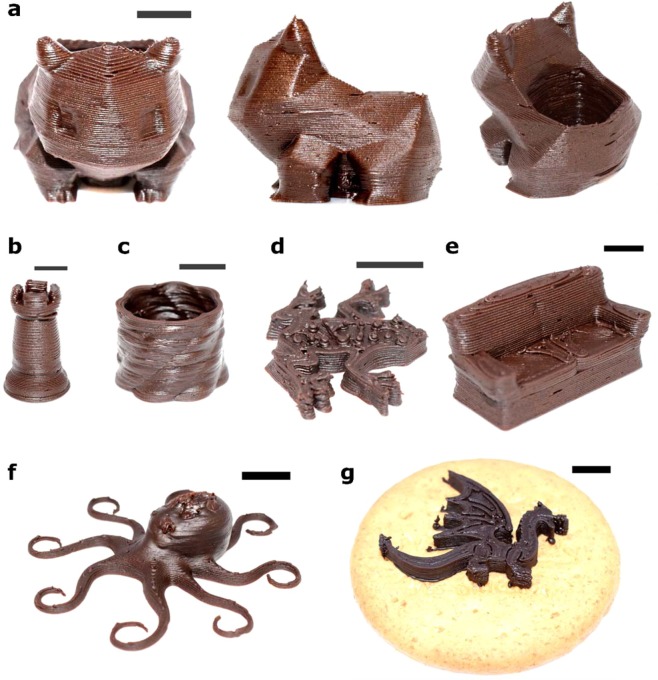
Optical images of 3D printed structures printed by direct chocolate-based ink 3D printing. (**a**–**f**) 3D structures of chocolate-based materials, printed with S20 and S25. (**g**) A 3D structure, printed with S20, of dragon directly printed on a biscuit as an underlying substrate. Scale bars = 1 cm.

With appropriate printing inks and operating parameters, we demonstrated fabrication of 3D models consisting of chocolate-based materials. The fabricated 3D structures of S20 and S25 are showcased (Fig. 6). These demonstrations highlighted the advantages of addition of cocoa powder to the inks in terms of (1) viscosity of the printing ink (ranging from 10^2^ to 10^4^ Pa.s) and (2) alteration of ink behavior from liquid-like to solid-like with yield stress. Unlike the hot-melt extrusion of chocolate 3D printing, all demonstrations in the current work was done at the room temperature without any heating elements. A Bulbasaur with a big cavity at the back was printed (front, side and top views in Fig. 6(a)). Different 3D geometries were directly fabricated (Fig. 6(b) to (f)) on the glass substrate. Using appropriate calibrations performed in the *z*-direction, we also performed Ci3DP on another edible substrate (*e.g.* a biscuit) (Fig. 6(g)). In the same way, 3D models consisting of P7.5 to P12 were printed (Supplementary Fig. S11). Overall, cocoa powders was used as an additive to modify the rheological properties of chocolate syrups and pastes. Characterization of their rheological properties ensured that the inks were appropriate to form self-supporting layers to form 3D structures. We foresee other materials with long polymer chains (such as starch and other carbohydrate derivatives) would serve as an effective additive for the formation of printable inks.

### Multi-material Ci3DP

**Figure 7:**
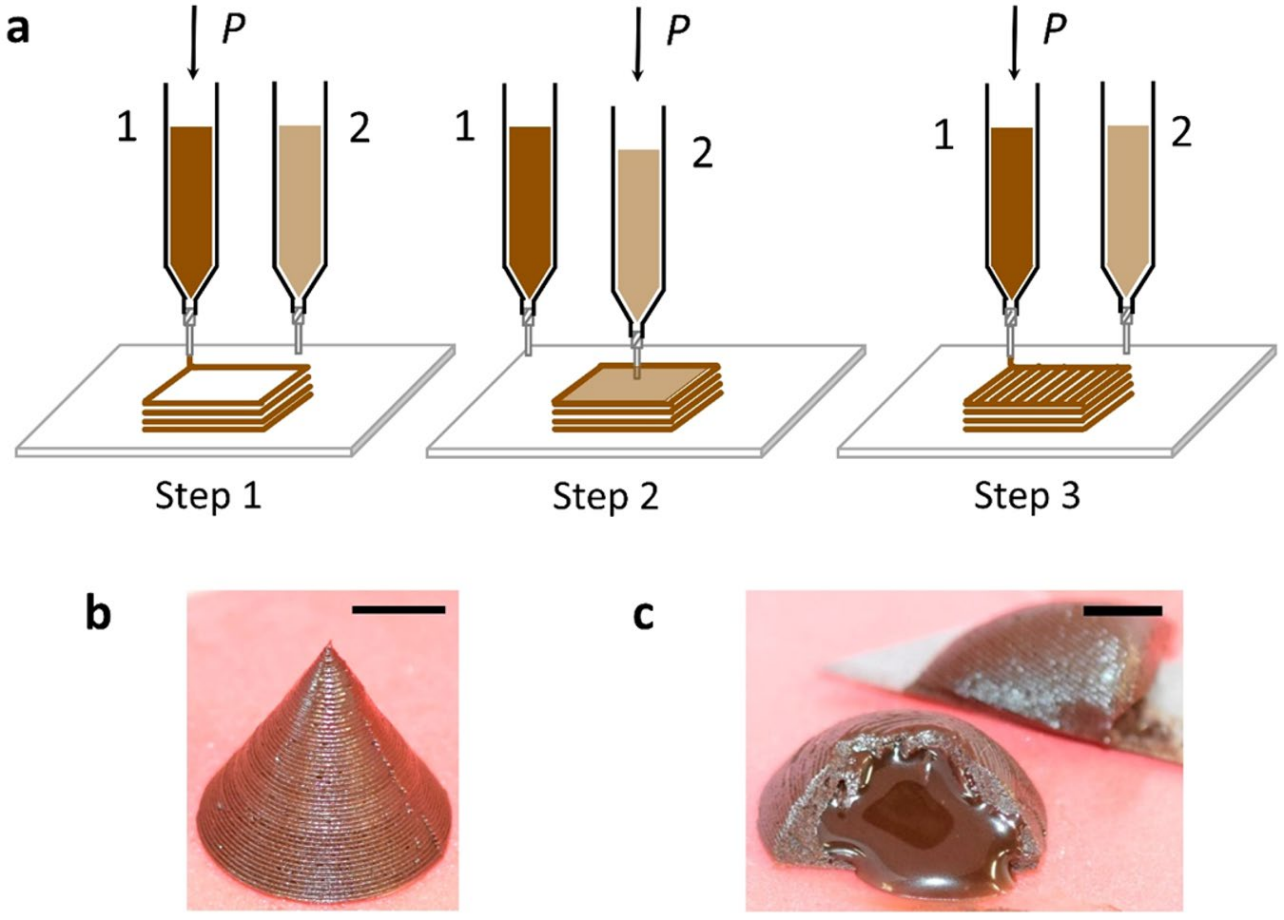
Multi-material food Ci3DP. (**a**) Schematic illustration of the steps involved in DIW of multi-food materials at room temperature. The sequence of steps allowed to print chocolate with an internal filling. (**b**) 3D structure of a cone-shaped enclosure containing a liquid chocolate syrup as a filling. (**c**) An optical image highlighting the liquid chocolate syrup flowing out of the 3D printed enclosure. Scale bars = 1 cm.

Finally, we demonstrated multi-material printing for Ci3DP using a DIW 3D printer equipped with two dispensing syringes (*i*.*e*. D1 and D2). We fabricated a 3D cone-shaped structure containing the liquid chocolate syrup as filling (Fig. 7). Figure 7(a) describes the steps to fabricate a 3D structure with filling. Briefly, a 3D cone-shaped enclosure was fabricated using one dispenser (D1), and the empty space within the 3D enclosure was filled with a chocolate syrup using another dispenser (D2). Finally, the remaining portion of the 3D enclosure was fabricated using D1. The final structure contained a liquid chocolate syrup as a filling (Fig. 7(b)). The inside filling can be observed once the enclosure was cut in two pieces (Fig. 7(c)). In this demonstration, we demonstrated that Ci3DP can be readily extended to multi-material food printing. Crucially, this work was enabled simple control over the rheological properties of the chocolate-based inks; two inks possessed different rheological properties at room temperature. The simplicity to prepare printable inks and fabrication of 3D structures at room temperature makes Ci3DP a unique method to perform multi-material food printing. Multi-material food objects consisting of range of liquid materials as fillings such as milk-based products (*e.g.* cream and yogurt) can be fabricated in a sequential manner using multiple dispensers. Ci3DP should be readily extended to fabricate multi-material food object with higher complexity and self-supporting properties conferred by the properties of the printing ink.

## Conclusions

In this paper, we presented a unique and simple method of direct ink writing (DIW) to fabricate complex 3D structures using chocolate-based materials at room temperature. The process was termed chocolate-based ink 3D printing (Ci3DP) to highlight the importance to formulate inks with adequate rheological properties for DIW. We formulated liquid inks consisting of chocolate-based materials (syrups and pastes) and different concentrations of cocoa powder. The characteristics of the chocolate-based ink were altered from a liquid-like fluid to a solid-like with zero-shear yield stress. The chocolate-based inks were shear-thinning and capable of forming self-supporting layers at room temperature. Our approach bypassed a major requirement of temperature control to perform 3D printing of chocolates by hot-melt extrusion. Ci3DP offered an easy route to fabricate 3D structures of chocolate-based inks with liquid fillings using multiple dispensers.

Ci3DP is flexible and should be capable of fabricating customized food in a wide range of materials with tailored texture and nutritional content. The effect of addition of varying concentrations of cocoa powders in chocolate-based inks on the textural, nutritional and sensorial properties are to be studied. This approach offers an alternative route of 3D modeling of food, especially when food ingredients or additives are sensitive to temperature. The use of multiple nozzles should offer interesting avenues to control distribution of the materials within the printed structure and shall find applications in the design of texture and controlled release of nutrients.

## Methods

### Preparation of inks

We tested three commercially available food syrups and pastes for their suitability of 3D printing. Cake Mate cake icing (Signature Brands, LLC., Florida, USA) was used as it is. The chocolate-based inks were prepared by mixing different concentrations of Hershey’s cocoa powder (Hershey, USA) in (1) Hershey’s chocolate syrup (Hershey, USA) and (2) Nutella hazelnut chocolate spread (Nutella, Singapore). The major ingredients of the cake icing, Hershey’s chocolate syrup and Nutella hazelnut chocolate spread are summarized (Table 1). The formulated chocolate inks were stirred continuously to be homogeneous and then stored in sealed bottles until used for printing.

**Table 1:** Major ingredients of the printing inks.

Product	Ingredients
Hershey’s chocolate syrup	High fructose corn syrup, corn syrup, water, cocoa, sugar
Nutella hazelnut chocolate spread	Sugar, vegetable fat (palm), hazelnuts, fat reduced cocoa powder, skimmed milk powder, whey powder
Cake icing	Sugar, vegetable shortening (palm, sunflower and hydrogenated cottonseed oils), water, corn syrup

### Rheological characterization

The measurements of the rheological properties were conducted using a rheometer (Discovery HR-2, TA Instruments, USA) with a 40 mm parallel plate. The gap between the plate and the stationary flatbed was 1000 *μ*m in all the rheological experiments. A spatula was used to deposit the ink carefully on the bottom plate. The top plate was lowered to the set gap of 1000 μm. The excess sample squeezed out between the plates was then removed neatly to avoid the edge effects. Similar loading procedure was followed for all the measurements. All experiments were performed at room temperature and under ambient pressure. Viscosity test were performed by applying a stepwise shear rate ramp from 0.01 to 2000 s^−1^. A measure of thixotropy of the inks was studied by thixotropy loop test. The applied shear rate was logarithmically increased from 0.0001 to 10 s^−1^ and then returned back to the initial shear rate at the same time interval. The shear stress versus the shear rate was plotted and the inbound area between upper and lower curve was measured as a measure of thixotropy. Storage modulus (*G*′) and loss modulus (*G*′′) were determined by oscillatory stress sweep test by applying sinusoidally varying stresses ranging from 0.1 to 3000 Pa with a constant frequency of 1 Hz. Variation of storage and loss moduli with respect to applied shear stress of the printing inks provided useful information about the sample microstructure. This test allowed determination of linear viscoelastic region (LVR) of the samples. All the data from rheological measurements such as viscosity, yield stress, elastic modulus and thixotropic loop area was reproduced five times and presented as average values with the standard deviation.

### DIW 3D printer

The setup of experiment used in this work is shown in the supplementary information (Supplementary Fig. S1). Inks were deposited using a DIW 3D printer using a commercial 3D printing robot and a dispenser (SHOTmini 200 Sx and IMAGE MASTER 350 PC Smart, Musashi Engineering Inc., Japan). The liquid dispenser was equipped with a single syringe that can be charged with inks with different formulations and was attached with a precise pressure controller (ML-5000XII and ML-808GX, Musashi Engineering Inc., Japan).

### Protocol for Ci3DP

The workflow used to fabricate the 3D printed objects in this study was as follows: MuCAD V software (Musashi Engineering Inc., Japan) was used to generate the design and printed using liquid dispensers. For 3D designs, STL models were obtained freely online (Supplementary Table S1). The STL models were then scaled down and sliced using the Slic3r software into 200–500 *µ*m thick layers to generate the G-code instructions. Then the G-Code was converted into MuCAD V recognizable format by a customized Python script and then sent to the printer. The inks were directly poured into cylindrical dispensing barrels for printing in Ci3DP. The nozzle was attached to the cylindrical ink barrel and placed into its respective position in the liquid dispenser. For every nozzle attached, calibrations in *xy*- and *z*- directions (nozzle-to-substrate distance) were performed. The pressure required for the extrusion, dispensing head velocity in each of three directions, dispensing head acceleration and deceleration times were calibrated according to the printing pattern to be fabricated and the viscosity of the ink. The chocolate-based inks were extruded through the attached nozzle to form a filament that was deposited onto the glass substrate in a series of layers. All the experiments were performed at room temperature.

### Imaging

Photographs were taken using a Nikon D5600 camera (Nikon, Japan) and Hirox digital microscope KH-8700 (Hirox Co Ltd., Japan) under white-light illumination. All image processing was done using ImageJ software^37^.

## Supplementary information

Supplementary Information

Supplementary Movie S1

Supplementary Movie S2
