# Supplementary material for: Chocolate-based Ink Three-dimensional Printing (Ci3DP)
Source: Sci Rep. 2019 Oct 2;9:14178. doi: 10.1038/s41598-019-50583-5 (PMC6775229; doi:10.1038/s41598-019-50583-5)
Supplement: Supplementary file 1 — Supplementary Information [file 41598_2019_50583_MOESM1_ESM.docx]

**Chocolate-based Ink Three-dimensional Printing (Ci3DP): Supplementary information**

Rahul Karyappa^1^ and Michinao Hashimoto^1,2,a)^

^1^ Digital Manufacturing and Design (DManD) Centre, Singapore University of Technology and Design, 8, Somapah Road, Singapore 487372

^2^ Pillar of Engineering Product Development, Singapore University of Technology and Design, 8, Somapah Road, Singapore 487372

^a)^Electronic mail: hashimoto@sutd.edu.sg

**
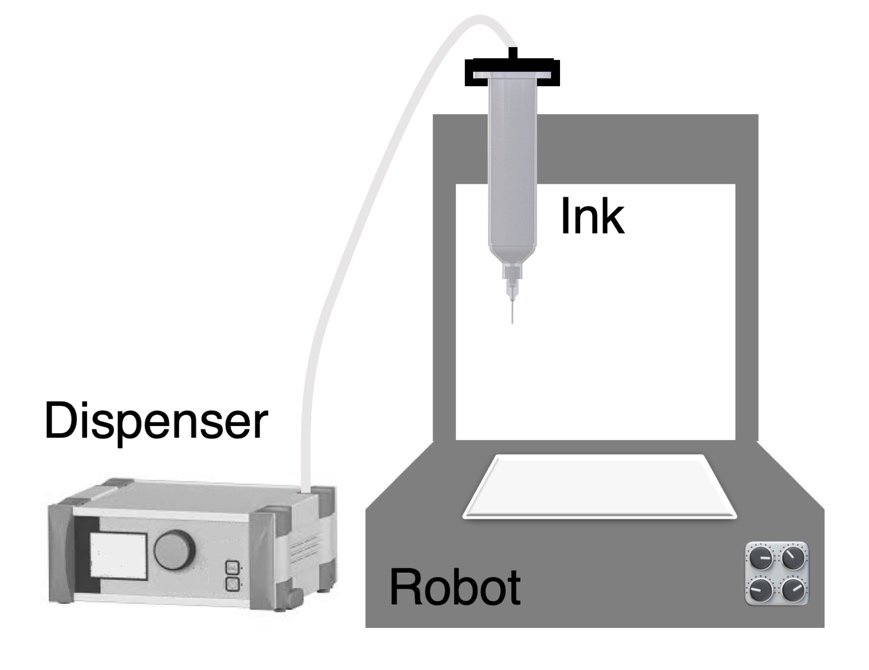
**

**Fig. S1.** Schematic of the direct ink writing (DIW) 3D printer and the pneumatic dispenser used in this study.


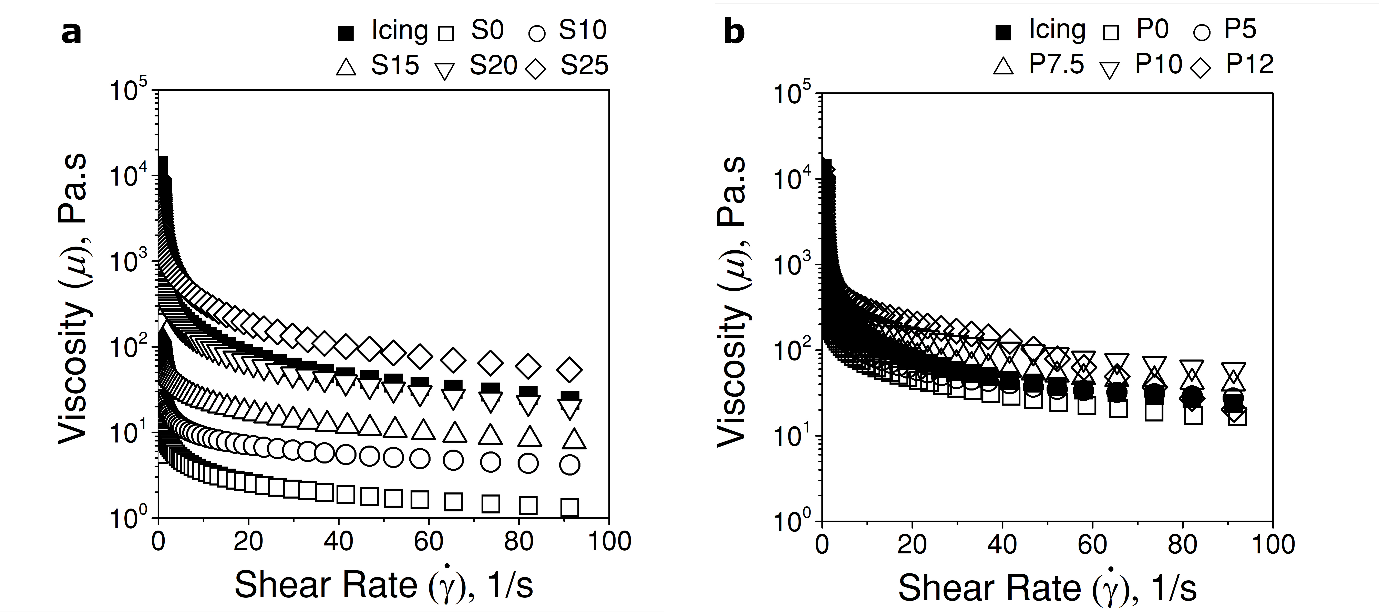


**Fig. S2.** Rheological characterization of chocolate-based inks and cake icing. Plots showing viscosity (*μ*) as a function of applied shear rate ($\dot{\gamma}$) for (**a**) the chocolate syrup with cocoa powders (S0 to S25) and (**b**) the chocolate paste with cocoa powders (P0 to P12).

**
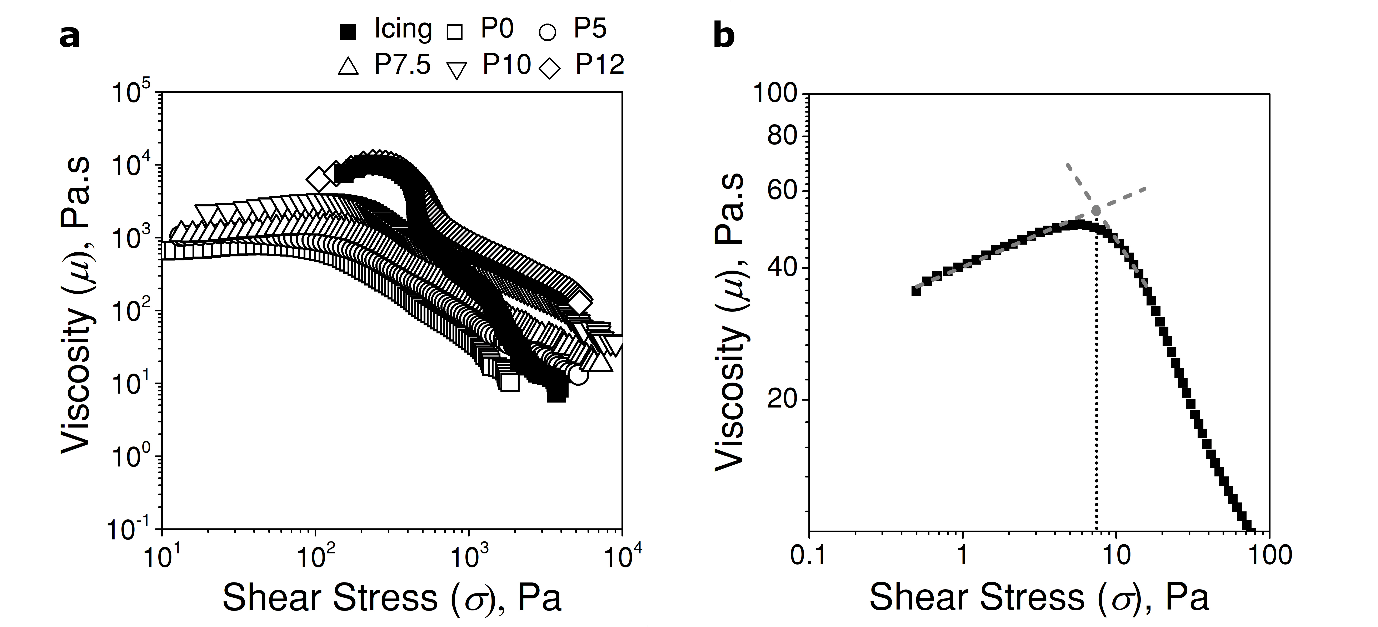
**

**Fig. S3. (a)** Plot of viscosity (*μ*) as a function of applied shear stress (*σ*) for the chocolate paste with cocoa powders (P0 to P12) and cake icing. (**b**) Determination of yield stress (*σ_y_*) from a plot of viscosity (*μ*) as a function of applied shear stress (*σ*) using the intersection of two tangent lines.

**
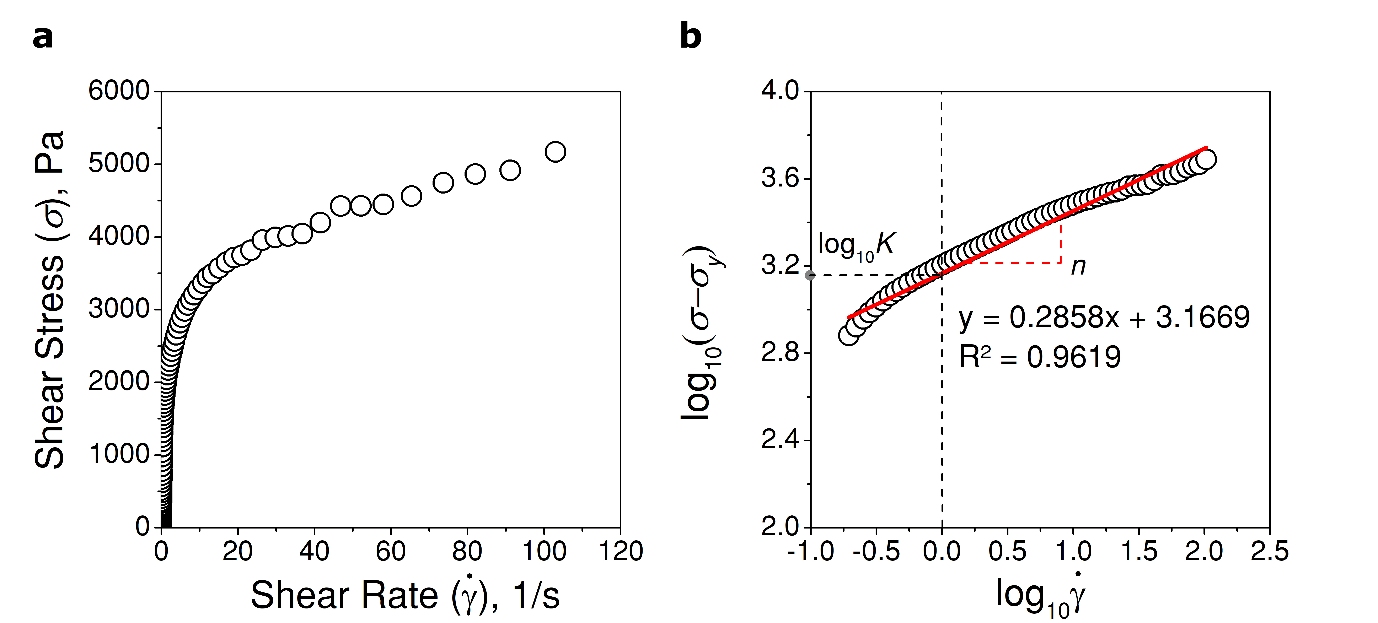
**

**Fig. S4.** (**a**) Plot of shear stress (*σ*) as a function of applied shear rate ($\dot{\gamma}$) for the syrup-based ink (S25). (**b**) Shear stress (*σ*)-shear rate ($\dot{\gamma}$) measurement of S25 fitted with Herschel-Bulkley (HB) model on a log-log scale.


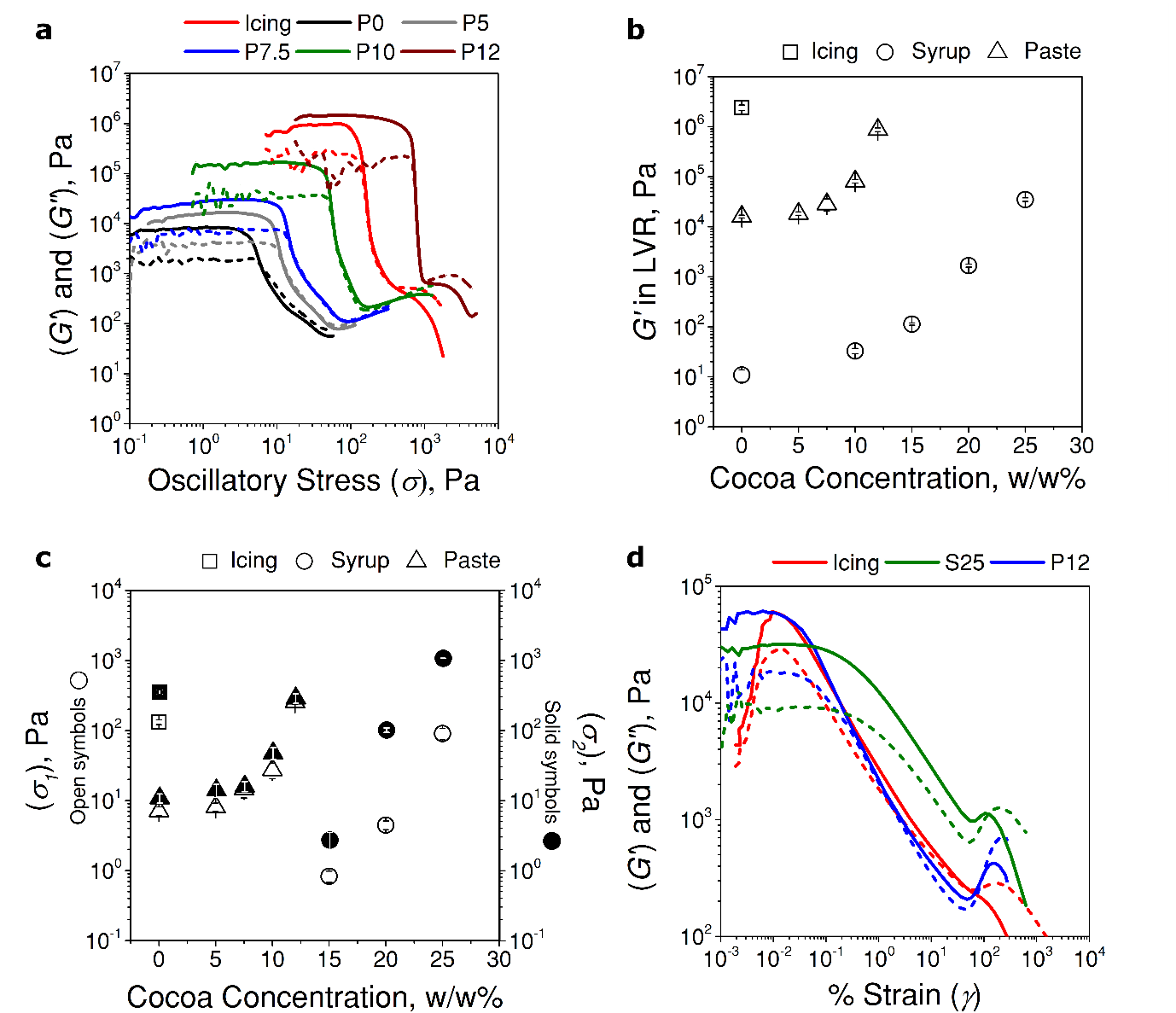


**Fig. S5.** Oscillatory stress measurements of chocolate-based inks and cake icing. (**a**) Storage (*G'*, solid lines) and loss moduli (*G''*, dashed lines) as a function of applied oscillatory shear stress (*σ*) for the paste-based inks. (**b**) Elastic modulus (*G'*) in LVR as a function of the concentration of cocoa powder in syrup (S0 to S25), paste (P0 to P12) and cake icing. (**c**) Comparison of two yield points (*σ_1_*: stress when *G'* deviates from LVR and *σ_2_*: crossover stress at *G' = G''*) as a function of concentration of cocoa powder in syrup (S0 to S25), paste (P0 to P12) and unmodified cake icing. (**d**) Storage moduli (*G'*, solid lines) and loss moduli (*G''*, dashed lines) as a function of % strain (*γ*) for S25, P12 and cake icing.


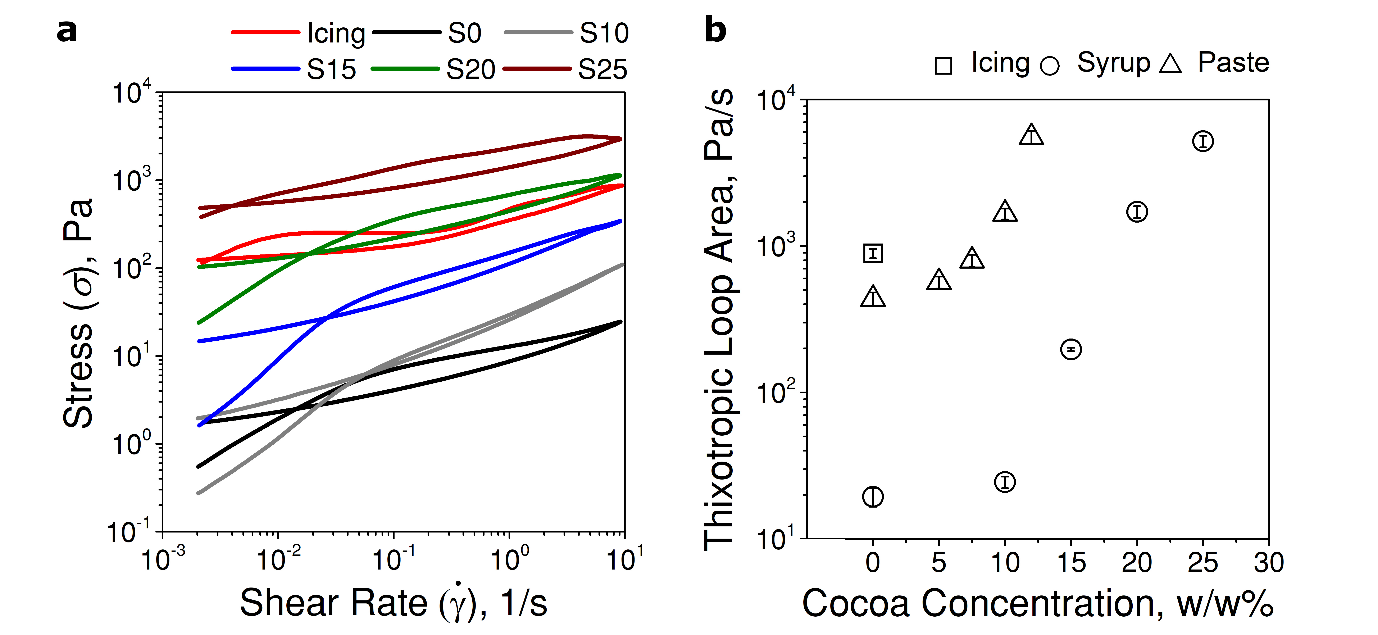


**Fig. S6.** (**a**)Plot showing the results of thixotropic loop test, shear stress (*σ*) as a function of increasing and decreasing shear rate ($\dot{\gamma}$), for the syrup-based inks and unmodified cake icing. (**b**) Thixotropic loop area as a function of concentration of cocoa powder in syrup (S0 to S25), paste (P0 to P12) and cake icing.

**
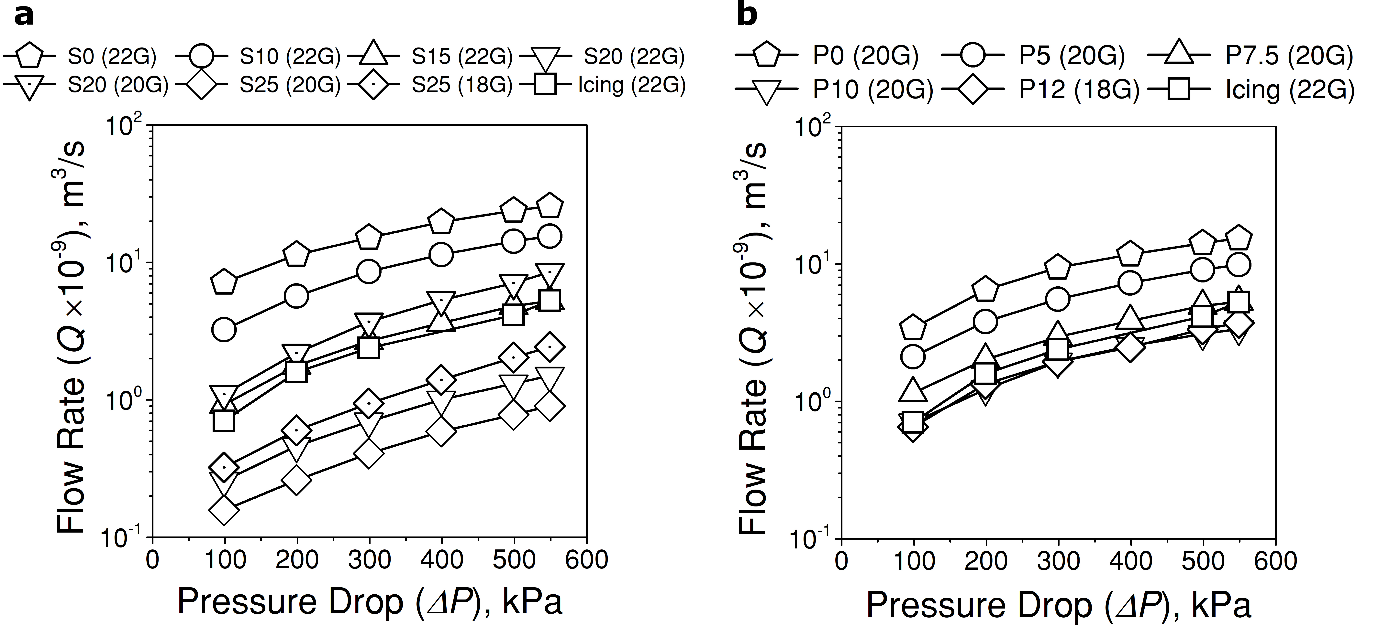
**

**Fig. S7.** Plots showing the rate of volumetric flow rate (*Q*) as a function of the pressure drop (*ΔP*) for (**a**) syrup-based inks (S0 to S25) and cake icing, and (**b**) paste-based inks (P0 to P12) and cake icing.

**
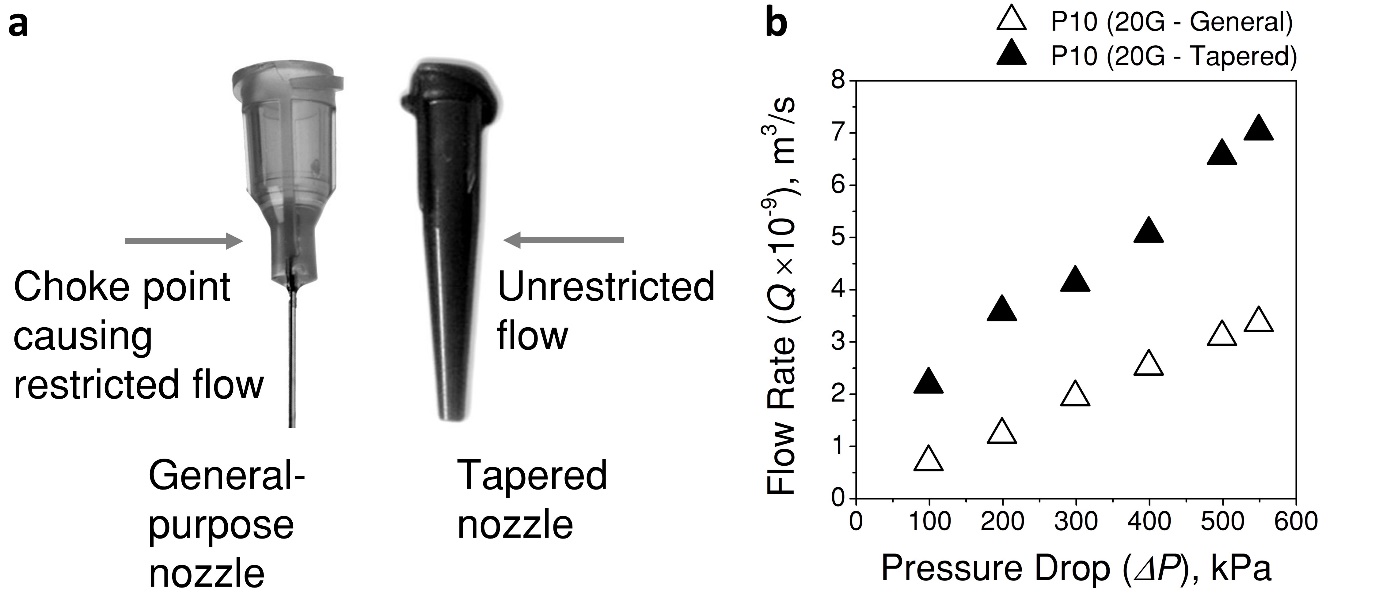
**

**Fig. S8. (a**)Types of nozzles used in Ci3DP. Optical images of a general-purpose nozzle and a tapered nozzle. (**b**) Plot showing volumetric flow rate (*Q*) as a function of pressure drop (*ΔP*) through a general-purpose nozzle (20 Gauge, *d* = 600 μm) and a tapered nozzle (20 Gauge, *d* = 600 μm) for P10.

**
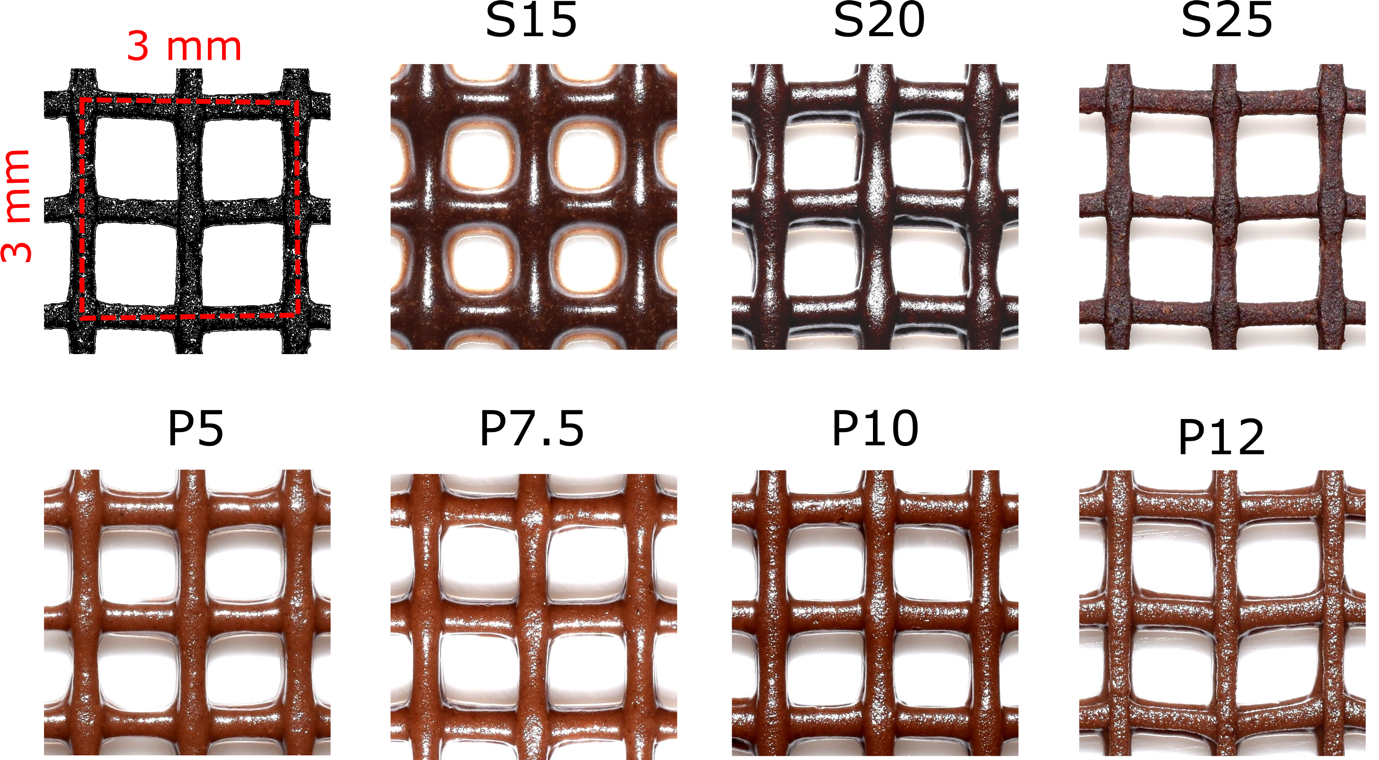
**

**Fig. S9.** Optical micrographs of the 3D mesh models fabricated with the syrup-based inks (S15 to S25) and paste-based inks (P5 to P12) at *t_p_* = 30 min (time after printing).

**
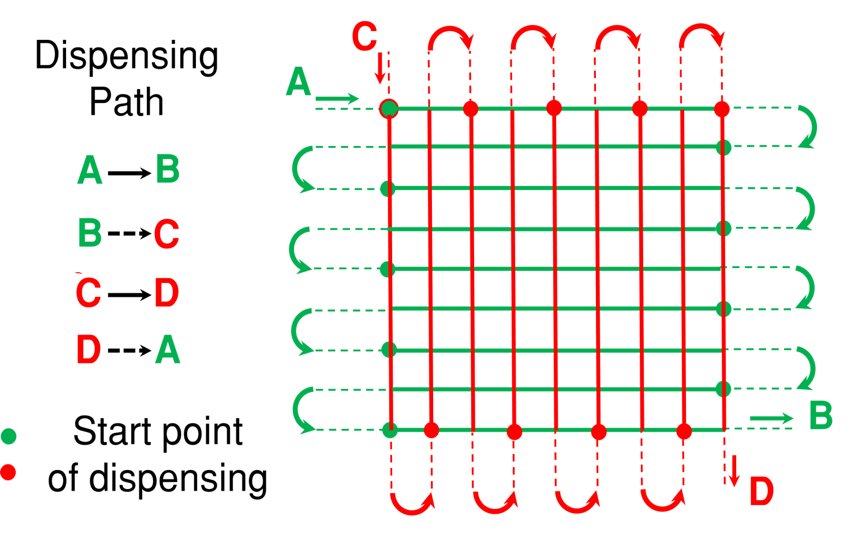
**

**Fig. S10.** Schematic illustration of the paths to fabricate 3D mesh. Along the solid lines, the dispensing pressure was applied, and along the dashed lines, the pressure was not applied. Direction of dispensing of the ink is shown with the arrows. Filled circles (colored in red and green) indicate the points of the start of dispensing (*ΔP* > 0). The first layer was formed by following the path of dispensing from A to B (lines in green colors). The nozzle then moved from B to C and the second layer was formed by dispensing ink from C to D (lines in red color) with a set value of *Δz*. 3D mesh was fabricated by alternating these two modes of printing until the desired number of layers of inks was printed.

**
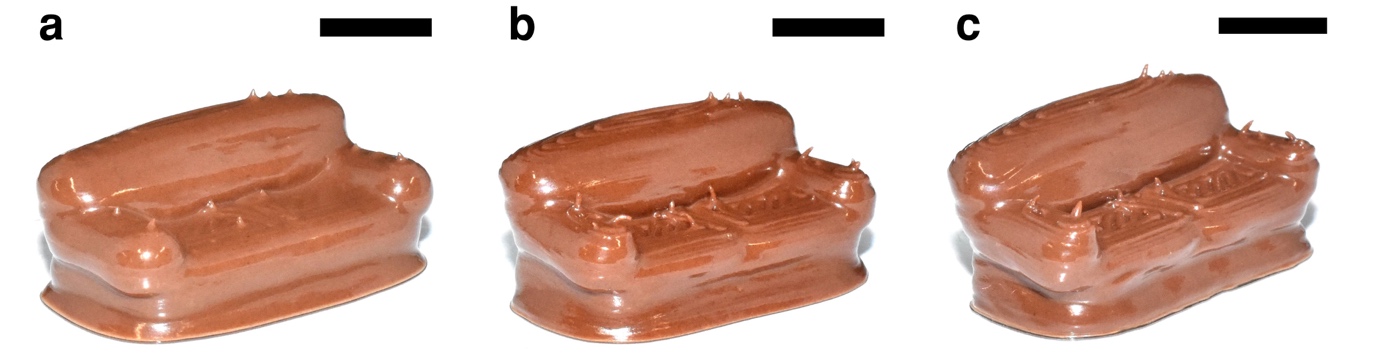
**

**Fig. S11.** Optical images of 3D structures consisting of paste-based inks printed by Ci3DP. (**a**) P7.5, (**b**) P10, and (**c**) P12. Scale bars = 1 cm.

**Table S1**: STL models obtained freely online.

| **STL models** | **Reference** |
| --- | --- |
| Knight (Fig. 3(d)) | File by tetralite, https://www.thingiverse.com/thing:378322 |
| Bulbasaur (Fig. 6(a)) | File by mike_new, https://www.thingiverse.com/thing:869668 |
| Rook (Fig. 6(b)) | File by tetralite, https://www.thingiverse.com/thing:378322 |
| Screw cup (Fig. 6(c)) | File by Misguided, https://www.thingiverse.com/thing:3270 |
| Frog (Fig. 6(d)) | File by muzz64,  https://www.thingiverse.com/thing:559427 |
| Couch (Fig. 6(e)) | File by Willie,  https://www.thingiverse.com/thing:141404 |
| Octopus (Fig. 6(f)) | File by Kempi05,  https://www.thingiverse.com/thing:776710 |
| Dragon (Fig. 6 (g)) | File by muzz64, https://www.thingiverse.com/thing:570345 |

**Supporting Movie S1.** Ci3DP of S10.

**Supporting Movie S2.** Ci3DP of S20.

**Modeling the flow of ink in Ci3DP.** The flow of the ink through the syringe and the nozzle, and the rebuilding time of the internal microstructure of the ink once it exits the nozzle was modelled by observing the change in *μ* with a change in $\dot{\gamma}$ by rheometry (Fig. 1(a, b)). The ink experienced low $\dot{\gamma}$ (< 1 s^-1^) in a syringe. In the nozzle, $\dot{\gamma}$ increased to the values of 10^2^ to 10^4^ s^-1^, depending on the flow rate. When the ink exited the nozzle, flow of the ink stopped. The ink quickly regained a large value of * *to form self-supporting layers. The change in *μ* suggested fast recovery of the microstructures of the chocolate inks, supporting the applicability of those inks in DIW 3D printing.

**Effect of *h* and* Δz* on fidelity of printing.** The distance between the vertical positions of the dispensed filaments affected the fidelity of printing of the 3D printed objects. The nozzle-to-substrate distance (*h*) was important for the stability of the extruded filament of the ink deposited on the substrate. The extruded filament of the ink on a stationary substrate undergoes liquid rope-coil instability when the distance exceeds a critical value^1^. When the nozzle or substrate are not stationary, the printing parameters need to be adjusted correctly. For the extruded filament coming out of the nozzle, three different conditions are possible based on the velocity of the extruded filament coming out of the nozzle (*v_i_*) and the velocity of the dispensing head (*v*): (1) *v* = *v_i_*, no instability and formation of a straight filament, (2) *v* > *v_i_*, stretching of the extruded filament which may either break or form a thinner straight filament, and (3) *v* < *v_i_*, undergo instability that produces meandering or coiling patterns^2,3^.

We initially performed print tests to determine the layer height (*Δz*) required to make 3D models. We identified three situations based on *ΔP*, as *v_i_* was increased when *ΔP* was increased. Firstly, when *v* << *v_i_* (*w* >> *d*, over-extrusion), expanded and flattened filament was obtained which resulted in dragging of the previously extruded ink by the moving nozzle. Secondly, when *v* ≤ *v_i_* (*w* > *d,* desired extrusion), slightly wide filament was obtained. Finally, when *v* > *v_i_* (*w* < *d*, under-extrusion), stretching or broken filament were obtained. The second situation was desirable in Ci3DP for the proper attachment of the adjacent layers; the extruded filament must spread on the printed layer to ensure the fidelity of the printing.

In a representative condition with the cake icing as printing ink (*μ* = 1.54 $\times$ 10^4^ Pa.s), we set (*ΔP*, *d*) = (150 kPa, 700 μm) to obtain *m* = 4.6 mg/cm. Under this condition, the deposited filament had the width of 1126 ± 24 μm and the height of 488 ± 12 μm. The layer height was set as *Δ z* = 400 μm (which was lower than the printed layer height) to ensure the adhesion between two adjacent layers of the printed chocolate-based inks to achieve good fidelity of printing. Similar optimization of the printing conditions was performed for each ink.

**Thixotropic loop test.** The thixotropic loop test consisted of two parts, an upward curve (first part) and a downward curve (second part). The effect of increased shear rate (first part) followed by decreased shear rate (second part) on shear stress was observed; the shear rate was increased from 0.0001 to 10 s^-1^ and then decreased from 10 to 0.0001 s^-1^ (Supplementary Fig. S6(a)). The shear stress increased with increasing applied shear rate due to the elastic response of the inks. As the shear rate was increased, the breakdown of the structures of the inks led the transition of the ink properties from elastic to viscous. The breakdown of the structures of the inks can be confirmed by the relative positions of the curves; the downward curve was located under the upward curve. As the shear rate was decreased, the recovery of the broken structures of the inks started. To evaluate the thixotropy, the inbound area between the upward and downward curve was measured^4^; the inbound area measures the energy required to break down the ink structure.

**References**

1 Habibi, M., Rahmani, Y., Bonn, D. & Ribe, N. M. Buckling of Liquid Columns. *Phys. Rev. Lett.* **104**, 074301 (2010).

2 Yuk, H. & Zhao, X. A new 3D printing strategy by harnessing deformation, instability, and fracture of viscoelastic inks. *Adv. Mater.* **30**, 1704028 (2018).

3 Passieux, R. *et al.* Instability-assisted direct writing of microstructured fibers featuring sacrificial bonds. *Adv. Mater.* **27**, 3676 (2015).

4 Barnes, H. A. Thixotropy - a review. *J. Non-Newtonian Fluid Mech.* **70**, 1-33 (1997).
